# What Do We Really Know about Cognitive Inhibition? Task Demands and Inhibitory Effects across a Range of Memory and Behavioural Tasks

**DOI:** 10.1371/journal.pone.0134951

**Published:** 2015-08-13

**Authors:** Saima Noreen, Malcolm D. MacLeod

**Affiliations:** School of Psychology & Neuroscience, University of St Andrews, Fife, United Kingdom; University of Tasmania, AUSTRALIA

## Abstract

Our study explores inhibitory control across a range of widely recognised memory and behavioural tasks. Eighty-seven never-depressed participants completed a series of tasks designed to measure inhibitory control in memory and behaviour. Specifically, a variant of the selective retrieval-practice and the Think/No-Think tasks were employed as measures of memory inhibition. The Stroop-Colour Naming and the Go/No-Go tasks were used as measures of behavioural inhibition. Participants completed all 4 tasks. Task presentation order was counterbalanced across 3 separate testing sessions for each participant. Standard inhibitory forgetting effects emerged on both memory tasks but the extent of forgetting across these tasks was not correlated. Furthermore, there was no relationship between memory inhibition tasks and either of the main behavioural inhibition measures. At a time when cognitive inhibition continues to gain acceptance as an explanatory mechanism, our study raises fundamental questions about what we actually know about inhibition and how it is affected by the processing demands of particular inhibitory tasks.

## Introduction

The notion that we might possess some degree of mental control over which particular memories can be brought to mind continues to excite the imagination of therapists, the media, and the public. Cognitive researchers engaged in understanding the basic mechanisms underlying human memory performance are similarly excited by the ramifications of such executive control for our understanding of human memory [1–8]. At both a theoretical and practical level, memory retrieval represents a particularly thorny problem to solve given that the retrieval cues typically employed to access particular items of target material from memory are often under-specified. This means that there is an in-built tendency to access not only the memory of interest but also other related but non-target memories which then compete for retrieval with the target material we wish to recall. Thus, one of the big questions in memory research is how this kind of retrieval problem is solved; how does memory resolve the issue of unwanted competition at retrieval?

According to an inhibitory or suppression account of memory retrieval [9–13] inhibitory resources are thought to be recruited in such a way that unwanted or irrelevant memories can be prevented from coming to mind, thereby promoting the retrieval of target material we wish to remember. In broad terms, this kind of suppression-plus-selection mechanism explains how, for the most part, we are able to achieve goal-oriented remembering [9, 11, 14–17].

One of the principal means by which this kind of inhibition has been explored is via the selective retrieval-practice procedure [10]. In this paradigm participants are typically presented with lists of category-exemplar pairs to study (e.g., *fruit-orange*, *fruit-apple…*.*; tree-sycamore*, *tree-oak…; drink-gin*, *drink-vodka…*.*; sport-soccer*, *sport-tennis*….). Following this initial presentation phase, participants are required to retrieve a subset of exemplars from some of the studied categories using the category name plus a letter stem as a prompt (e.g., *tree-sy____; sport-te___*_). Participants are then presented with an unrelated interpolated task lasting ~5–20 mins, followed by a final recall test for all the items originally presented.

In addition to the facilitated recall performance for those items that receive retrieval practice, systematic forgetting effects also typically emerge for unpractised items from practised categories (i.e., Rp- items) compared to baseline items (i.e., non-practised items from non-practised sets, Nrp items). This phenomenon is particularly noteworthy because, in a sense, both Rp- items and Nrp items are treated in a functionally identical manner; that is, neither receive any additional retrieval practice, and yet, Rp- items are retrieved more poorly than Nrp items. This recall impairment is referred to as ‘retrieval-induced forgetting’ and has been demonstrated across a wide range of knowledge domains [11, 18–25].

Anderson and colleagues [9–11, 26–29] have argued that one of the most compelling reasons as to why this kind of forgetting should be attributed to inhibition rather than non-inhibitory associative blocking is because Rp- items remain forgotten even when independent cues have been employed at final test (i.e., novel cues). The logic behind the use of such independent cues is that, if the forgetting of Rp- items is simply a function of some form of associative interference or blocking between the cue and its exemplar, the provision of a novel cue at final test (i.e., one that has not been used in the original presentation phase) should circumvent any such interference [4, 9, 11, 30].The fact that forgetting effects for Rp- items persist despite the use of such independent cues is consistent with the notion that the memory representation itself is no longer available to conscious inspection; that is, it has been inhibited.

More recently, this rationale has been challenged. Specifically, it has been argued that the independent cues used in retrieval inhibition studies are not truly independent and may, in fact, aid participants to cue original target words by accessing other semantically-related cues (see [31–33] for alternative accounts). Although Huddleston and Anderson [28] have demonstrated that such enhancement effects only arise where semantic relationships have not been adequately controlled, there remains the practical difficulty of how to determine whether cue semantic relatedness has been adequately controlled.

Perhaps anticipating such complexities, others have sought to devise new paradigms which provide additional support for an inhibitory account of retrieval-induced forgetting. Storm, Bjork, Bjork and Nestojko [34] developed the ‘impossible’ retrieval task procedure in which participants are cued for exemplars that are presumed to not actually exist and therefore impossible to retrieve. Storm and colleagues showed that the attempt to retrieve–even when unsuccessful–is sufficient to produce retrieval-induced forgetting of related competing material. They argued that it is the retrieval attempt rather than successful retrieval per se which causes the forgetting of competing items. It follows, therefore, that the forgetting of Rp- items in the impossible retrieval practice paradigm cannot be a function of blocking caused by the strengthening of Rp+ items as there were no Rp+ items to recall (see also [4, 29]).

An inhibitory framework has also been widely adopted by those interested in the notion of ‘memory stopping’- the apparent ability to prevent particular memories coming to mind [35–38]. This phenomenon has been extensively explored via the Think/No-Think (TNT) paradigm (a variant of the Go/No-Go task, [39]). Devised by Anderson and Green [35], the TNT paradigm captures the type of memory inhibition that arguably occurs when we are confronted with a strong reminder of a particular memory we would prefer to forget. In this paradigm, participants are asked to learn a list of unrelated word pairs (i.e., cue word-target word) to a specified criterion. Participants are presented with the original cue words and are then required to ‘respond’ or ‘suppress’ the target word associated with the cue; that is, in the ‘respond’ condition, participants are asked to remember the associated target word whereas, in the ‘suppress’ condition, participants are asked to keep the associated target word from entering conscious awareness.

Using both the same cue and independent cue techniques at final test [11], Anderson and Green [35] found that participants could successfully prevent unwanted target memories coming to mind and that the extent of this forgetting increased with the number of suppression attempts. These TNT forgetting effects have been replicated with a wide range of materials [36, 40–45] including, most recently, autobiographical memory [6, 7, 46] but see [47, 48] for alternative theoretical explanations for this kind of forgetting.

Some researchers have also sought to draw parallels between TNT forgetting and the ability to inhibit inappropriate pre-potent behavioural responses, suggesting that the same basic mechanism may be involved in the control of both memory and behaviour. Specifically, it has been argued that both the suppression of items from memory and behavioural stopping engages a common neural architecture, and that the apparent overlap between memory and behavioural systems means that one could expect to find generalized inhibitory control deficits in the population [3, 37, 38, 49]. Assuming this to be the case, it would be reasonable to expect that there may be a positive relationship between one’s ability to prevent unwanted thoughts or memories coming to mind, and one’s ability to override inappropriate pre-potent behavioural responses.

Following this line of investigation, researchers have begun to explore the relationship between behavioural and memory inhibition [50, 51]. Behavioural inhibition, as indexed by the stop signal, has been found to be correlated with performance on both the retrieval practice task [52] and the TNT task [53]. These findings are consistent with the notion that the inhibitory processes involved in the TNT and retrieval practice tasks may be analogues to inhibitory processes involved in behavioural response inhibition. In saying this, however, it is important to note that such studies to date have used only the stop signal task. Thus, it remains possible that the observed relationship between these tasks may not reflect inhibition per se but rather some other component of the stop signal task. In order to eliminate this possibility, therefore, it would be important to further explore the relationship between memory and behavioural inhibition using other kinds of behavioural inhibition tasks.

More fundamentally, one of the difficulties in accepting any kind of inhibitory account of forgetting is that the ascription of memory inhibition necessitates inferences being drawn about its existence on the basis of the *absence* of some other entity; that is, inhibition itself cannot be directly observed. Thus, despite the fact that an inhibitory account can often provide a more parsimonious account of the forgetting phenomena under investigation, inhibition as an explanatory mechanism remains susceptible to the primacy of non-inhibitory challenges [12, 54–57]. In addition, the use of the terms ‘suppression’ and ‘inhibition’ have suffered from past associations with other theoretical and therapeutic traditions which are often poorly understood and articulated in the cognitive literature (see [58, 59] for a discussion).

A notable exception to this has been the relatively well-specified inhibitory accounts of retrieval inhibition put forward by Anderson and colleagues [1, 10, 11, 13, 35, 60]. This detailed account, in turn, has prompted others to consider how such inhibitory effects may be represented at a computational level [16, 17, 61, 62]. Thus, to the casual observer, it might seem reasonable to conclude that our understanding of inhibitory control is relatively well-advanced but a number of fundamental issues remain to be resolved.

Primary amongst these unresolved issues is the nature of the relationship *between* the inhibitory forgetting effects observed in TNT and retrieval practice paradigms. Assumptions are often drawn about the nature of retrieval inhibition without the empirical justification for doing so. A common assumption, for instance, is that the forgetting effects observed in these paradigms are integrally related to one another because they are inferred to represent the same theoretical construct; that is, retrieval inhibition. Assuming this to be the case, one could (as a starting point) reasonably expect that the extent of forgetting observed on one inhibitory task would be correlated with performance on another. Indeed, reviews of this literature often discuss inhibitory control in terms of it constituting a unitary entity. As a result, different memory tasks are often used interchangeably and convergently to assess inhibition [9, 12, 21, 38, 49, 63]. Indeed, much of the literature on retrieval inhibition proposes that although the processes by which inhibition is invoked may be different in each paradigm, the goal-directed nature of forgetting is the same in each case. This classical conception of retrieval inhibition is similarly shared by those researchers who refute the notion of retrieval inhibition as an explanatory mechanism for the forgetting effects observed in retrieval practice and Think/No-Think paradigms [33, 54, 64–67].

Overlying this complexity is the role of individual differences in cognitive performance [68]. Memory inhibition would seem to be no different in this respect given that some people are better able to inhibit irrelevant or unwanted memories than others [3, 7, 69–71]. In addition, there are indications that this kind of inhibitory control diminishes with age [1, 72, 73] (although see [74]). Yet, it could be argued, that even if we were to take individual differences into account, the underlying assumption remains that performance across these various tests of memory inhibition should remain consistent; that is, even if an individual is starting from a low level of inhibitory control, one could expect that ability to be reflected in low levels of inhibitory forgetting across those tasks that purport to measure retrieval inhibition. Similarly, we could expect a consistent pattern of forgetting to emerge for those individuals with high levels of inhibitory control; that is, high levels of inhibitory forgetting on one task should be reflected in high levels of inhibitory forgetting on another task. Thus, notwithstanding the fact that marked individual differences may exist in our ability to inhibit memories, we could nonetheless expect performance to be correlated across those memory tasks that purport to provide a measure of inhibitory forgetting.

In the current article we consider a more nuanced interpretation in which we explore the possibility that the processing demands of these various memory tasks may be qualitatively different and, as a result, the pattern of inhibitory effects produced may, in fact, vary across tasks. The level of inhibitory control required effectively to suppress a highly trained association in the TNT task may be of a much higher order than the inhibitory resource required to suppress Rp- items in retrieval-induced forgetting studies. While this possibility has already been raised elsewhere [1, 74, 75], we are unaware of any systematic attempt to establish if this is the case. In the current study, we take the TNT and retrieval practice paradigms as proxies for high and low demand tasks, respectively.

One key difference in processing requirements between these various memory inhibition tasks is that, in the retrieval-practice paradigm, inhibition can be argued to reflect a passive mechanism whereby the activation of selected material causes the deactivation of competing material [10, 11, 14]. In the TNT task, in contrast, inhibition may reflect a more dynamic process whereby individuals are required intentionally to engage in suppression in order to keep particular memories from conscious awareness [35]. This crucial difference has led some researchers to propose that intentional forgetting tasks may place greater demands on the inhibitory mechanism and that this, in turn, may reflect a mode of controlled inhibition that differs from the kind of automatic inhibition thought to typically accompany selective retrieval practice [1, 74, 75]. If such a difference exists between controlled and automatic inhibition, we could expect no significant correlations to emerge in performance between retrieval practice and TNT tasks. Alternatively, it could be argued that forgetting in both the retrieval practice and TNT tasks operates via the degradation of the memory representation [76]. If so, we could expect that performance would be positively correlated across retrieval practice and TNT tasks.

In the current article, we provide the first empirical test of these various predictions. Employing a within-subjects design, we compared recall performance across the two principal means of measuring the inhibitory control of memory; that is, the ‘impossible’ retrieval practice procedure [34], and the TNT paradigm [35]. We also compared performance on a number of widely employed measures of behavioural inhibition (i.e., Stroop colour naming task, [77, 78]; and the Go/No-Go task[39]). In doing so, our study seeks to provide much-needed clarity about the kinds of relationships that occur across these various measures of inhibition and thereby lay the groundwork for a more comprehensive framework by which research on memory and behavioural inhibition can be evaluated and integrated.

## Method

### Participants

Eighty seven undergraduate students (28 male and 59 female) from the University of St Andrews, Scotland (mean age = 19.5 years; *SD* = 1.9) volunteered to take part in all three sessions of the study in exchange for course credit or the sum of £15 (~ $25.18 equivalent). Each of the sessions took place approximately 7–10 days apart. Participants were screened for any history of depression using a screening questionnaire, and current levels of depressive symptomology were measured using the Beck Depression Inventory-II [79]. All participants were right-handed, medication-free, and had normal or corrected-to-normal vision.

### Ethical Statement

Full ethical approval from the University of St Andrews Research Ethics Committee (UTREC) was obtained for the research. As all participants were over the age of 18 years, written informed consent to participate in the study was obtained from participants. In the consent forms, participants were asked to tick a box and sign if they were happy to participate in the experiment. Participants were also given the opportunity to ask any questions about the study prior to signing the consent form. Consent forms and the consent procedure were approved by the University of St Andrews Research Ethics Committee.

### Study overview

The study comprised the following sessions. In Session A, participants completed the TNT task [35]. In Session B, participants were given the ‘impossible’ retrieval-practice task [34], and in Session C participants were given the Stroop task (adapted from [80]), plus the Go/No-go task [81]. The order of sessions was counterbalanced throughout. Furthermore, in the session in which participants were required to complete more than one task, the order of presentation of the tasks was also fully counterbalanced.

### Memory Inhibition Tasks

#### Think/No-Think (TNT)

The TNT task comprised 36 neutral noun-noun word pairs taken from Anderson and Green (2001). These word pairs were divided into 3 sets of 12 with each set assigned to a respond, suppress, or baseline condition. The assignment of each set to each condition was fully counterbalanced across all participants. Furthermore, an additional 21 neutral noun-noun word pairs (also taken from [35]) were used as fillers.

Participants were presented with each word pair for 5000ms followed by a 500ms inter-trial interval. Participants were instructed to study each word pair as they would be tested on these words pairs immediately after the whole list had been presented. All 57 word pairs were presented, which included the 36 critical word pairs from the 3 sets plus 17 neutral-filler word pairs. Furthermore, 2 additional neutral filler word pairs were included at the beginning of the first block and 2 neutral filler word pairs were included at the end of the final block in order to eliminate primacy and recency effects. The neutral filler words remained the same across all participants.

Once all the word pairs were presented, participants were given a cued recall test. Each cue word was presented on the screen for a maximum period of 4000ms. Participants were asked to recall out loud the corresponding target associated with the cue word as quickly as possible. This was then followed by a delay of 300ms. Feedback (i.e., the correct response) was then provided for 1000ms followed by an inter-trial interval of 300ms. All participants were required to achieve a minimum of 75% on the assessment in order to continue with the procedure. This performance level ensured that participants showed a high level of remembering for the critical as well as the filler cue-target word pairs. Participants were given a maximum of 3 tests in which to achieve criterion. If participants did not reach criterion after 3 attempts, the experimental procedure was terminated and participants were debriefed and informed that their data would be safely discarded. In the current study, however, all participants reached criterion within the permitted number of attempts and therefore no participants were eliminated from the analysis.

In order to ensure that participants understood the procedure for the main suppression phase of the TNT task, participants were given a training phase which was exactly the same as the main suppression phase, but differed only in terms of the words used. Specifically, participants were informed that they would see some cue words, all of which they had seen previously. The cue words were presented either in red or green font. Participants were told to retrieve the associated word when presented with a green cue (‘respond’ condition) and to avoid saying or thinking about the corresponding word when presented with a red cue (‘suppress’ condition). In addition, in order to limit the range of possible strategies they could use, participants were told that they should not try to block the memory from coming to mind by replacing it with other memories or thoughts. Rather, participants were told that they should focus on the cue word and if the memory entered awareness to consciously push it out.

Five filler cue words appeared in green (two fillers appeared three times each, two fillers appeared four times each, and one filler appeared five times) plus 4 cue words appeared in red (two fillers appeared three times, one filler appeared four times and one filler appeared six times), thereby resulting in 35 trials.

Each trial began with a small cross appearing on the screen for 200ms. Subsequently, a cue word appeared for 4000ms. On respond trials, participants were instructed to recall aloud the target word. Incorrect responses resulted in the correct target being displayed for 1000ms in blue. This was then followed by an inter-trial interval of 400ms before the next trial began. On a suppression trial, participants were required to withhold their response and to prevent the corresponding word from coming to mind. Participants were given a total of 432 trials, which included respond and suppress words being presented 16 times each plus filler words being presented 8 times each. The 432 trials were split into four blocks of 108 trials, and participants were given a 60-second break following each block.

In the final test phase, participants were presented with both the same-probe cued recall test and an independent-probe test. Test administration order was counterbalanced. In the same-probe test, participants were presented with all 36 cue words. Participants were asked to disregard previous instructions and to recall all original target words associated with every cue. Each trial began with a cross being displayed for 200ms. Subsequently, a cue word was presented for 4000ms. Participants were asked to recall the associated target word for the cue. This was followed by an inter-trial interval of 400ms before the next trial began.

The independent-probe test also involved testing memory for all 36 target words. In this task, however, participants were presented with the first letter and the semantic category of all the target words and were informed that all the target words had been seen by them previously. Each trial began with a cross being displayed for 200ms. Subsequently, the first letter and the semantic category of the target word were presented for 4000ms. Participants were asked to recall the target word out loud. This was then followed by an inter-trial interval of 400ms before the next trial began.

#### ‘Impossible’ Retrieval Practice task

We chose to employ a version of the retrieval practice task devised by Storm and colleagues [34] because it arguably provides more compelling evidence for an inhibitory account of forgetting than the standard retrieval practice procedure (see also [82]). According to an inhibitory account, potential competing information is suppressed in order to facilitate the retrieval of target information. This occurs regardless of whether the retrieval of the target information has been successful or not [4, 82]. An interference account, in contrast, would predict that the impairment of competing material is actually a consequence of the target information being strengthened through retrieval practice. Thus, the rationale for using Storm and colleagues’ version of the retrieval practice paradigm is that, if the retrieval attempts are not successful, then the target of those retrievals cannot be strengthened and therefore no retrieval-induced forgetting (RIF) effect should emerge if the forgetting typically observed in retrieval practice studies is a function of blocking. The fact that Storm and colleagues found RIF effects even when retrieval practice was impossible provides further evidence in support of an inhibitory account of forgetting. Furthermore, given that Storm and colleagues found that RIF effects for possible and impossible target words were correlated with one another, we also sought to establish whether performance on both impossible and possible retrieval practice were distinctly correlated with other measures of inhibition.

Following the procedure outlined by Storm and colleagues [34], 48 category-exemplar pairs (consisting of six members of eight categories) were presented to participants. Each category was divided into two sets of three exemplars, resulting in 24 category-exemplar pairs for each list (i.e., three members of eight categories). From the eight categories, four categories received retrieval practice. Two categories were part of the possible retrieval practice and two categories comprised the impossible retrieval practice. In the *possible* retrieval practice condition, participants were presented with a category plus two letter stem cue for each of the category members and were instructed to retrieve the exemplars (note that these exemplars had not actually been presented in the study phase) (e.g., Fruit-Or___ for Fruit-Orange). In the *impossible* retrieval condition, participants were again shown a category plus two letter cue and instructed to recall the exemplars (e.g., Metal-Mi__). In this particular case, participants were asked to try to recall exemplars that did not actually exist for that category. In order to ensure that both possible and impossible retrieval conditions were matched as closely as possible, no category-exemplar pairs appeared in both the studied list and the retrieval practice task.

Participants were presented with one of the two sets of category-exemplars (each set contained 8 categories comprising 4 baseline categories, 2 possible retrieval practice categories, and 2 impossible retrieval practice categories). Both sets of category-exemplars were counterbalanced, so that half of the participants received one set of word pairs, and the other half received the other set of word pairs. In the study phase, participants were presented with each category-member pair for 5000msec and were instructed to study each word pair. This was then followed by an ISI of 300ms before the next category-member was presented. All 24 category-exemplar pairs were presented in random order but ensuring that no two exemplars from the categories were presented consecutively.

The retrieval practice phase consisted of 12 category-exemplars (6 possible and 6 impossible) presented three times each in random order, thereby resulting in 36 trials. The categories associated with possible or impossible retrieval practice were counterbalanced across participants, with no two stems sharing the same initial two letters, and also no two exemplars in a category sharing the same initial letter. Participants were given a response sheet and informed that they would see a series of category-plus-stem cues appearing on screen, one at a time for 5000ms each. They were also told that when the category-plus-stem cue appeared on screen, their task was to complete the two-letter stem on the response sheet provided. They were also told that there would be repetitions and that the pairs may or may not come from the list studied previously.

Once the retrieval practice phase had been completed, participants were provided with a 10-minute unrelated distractor task. The distractor task used was a modified version of the Trail Making Task, Version B [83] which involves presenting participants with a piece of paper which contains a series of numbers and letters. Participants were instructed to draw a single line sequentially going from number to letter (e.g., drawing a line from 1 to A and then 2 to B and so on). Although the Trail Making Task has been used as a measure of visual attention [84], in the current study, this task was used simply as an unrelated interpolated task.

The final recall test phase involved participants recalling all 24 category-exemplar pairs presented during the initial study phase. Participants were presented with a sheet of paper containing 24 category-plus-one-letter-stems presented in a random order. The presentation of the category-plus-one-letter-stems was counterbalanced across all experimental conditions. Participants were given 3 minutes in which to complete as many stem cues as possible.

### Behavioural Inhibition Tasks

Stroop-Colour Naming task (adapted from [80]) demands selective attention and provides a measure of the effect of interference and facilitation on a colour identification task using neutral, congruent and incongruent words. Incongruent words were defined as colour words that differed from the colour expressed by the word’s semantic meaning (e.g., the word ‘*red’* printed in ‘*green’* ink). Congruent words were defined as the print colour corresponding to the colour expressed by the word’s semantic meaning (e.g., the word ‘*red’* printed in *‘red’* ink). Neutral words were defined as words in which the semantic meaning did not relate to the colour expressed (e.g., the word ‘*table’* in ‘*orange’* ink). Participants were presented with a random series of 96 words (48 neutral, 24 congruent, and 24 incongruent). The words were presented in either red, or green, or orange, or blue ink. Participants were required to press the button on the keyboard that corresponded to the colour of the ink of a word whilst ignoring the semantic meaning of the word.

To determine if there were any group differences on selective attention, both correct and incorrect responses were recorded as well as the error rates for colour incongruent words. Furthermore, participants’ mean reaction times for neutral, congruent and incongruent words were calculated. To account for overall speed differences between participants in the Stroop task, interference and facilitation ratios were also analysed. The interference effect was expressed as the difference between the time needed to name the colour of a neutral word and the time taken to name the colour of an incongruent colour word (i.e., [incongruent–neutral] / neutral * 100). The facilitation effect was expressed as the difference between the time needed to name the colour of a neutral word and the time taken to name the colour of a congruent colour word (i.e., [congruent–neutral] / neutral * 100).

Go/No-Go task [81] was employed in order to provide a measure of behavioural inhibition. In this task, participants were presented with a red (target) or a green (non-target) circle in the centre of a computer screen. Participants were told to press the space bar as quickly as possible once they had seen the green circle (i.e., target stimuli ‘go’ cue) but to withhold responses to the red circle (i.e., non-target stimuli ‘no-go’ cue). The task consisted of four blocks. Each block contained 96 stimuli, which consisted of 72 (75%) green circles (‘go’ cues) and 24 (25%) red circles (‘no-go’ cues). This resulted in a total of 288 ‘go’ trials and 96 ‘no-go’ trials. Random trial order ensured that participants could not anticipate the presentation of ‘no-go’ trials. Stimuli were presented in the centre of the screen for 500ms, followed by an inter-stimulus interval (ISI) which ranged from 1250 to 1750ms (mean per block = 1500ms). The ISI was pseudo randomised to discourage anticipatory responses. Participants were given a 3-minute break after each block.

The number of commission errors on ‘no-go’ trials across all four blocks was the primary measure of behavioural inhibition on the task. Commission errors were defined as the total number of times participants responded (i.e., pressed the space bar) to ‘no-go’ cues. Furthermore, omission errors for ‘go’ trials were also calculated. Omission errors were defined as the total number of times participants withheld responses on the ‘go’ trials. Moreover, mean reaction times for participants’ responses during go trials were also calculated.

## Results

### Memory Inhibition Tasks

#### Think/no-think task

The dependent measure of interest here is the percentage of ‘respond’, ‘suppress’, and baseline items recalled correctly on both the same-probe and independent-probe tests. For the same-probe test, a one-way ANOVA showed a main effect of instruction, *F* (2, 84) = 21.97, *p*< 0.01. Subsequent pairwise analyses revealed a positive control effect with participants recalling significantly more respond than baseline words (*M* = 91.76, *SD* = 13.05 vs. *M* = 83.24, *SD* = 17.31; *t* (86) = 3.30, *p*< 0.01, d = 0.55). Furthermore, our analyses revealed a negative control effect with participants recalling significantly fewer ‘suppress’ words than baseline (*M* = 74.90, *SD* = 19.33 vs. *M* = 83.24, *SD* = 17.31; *t* (86) = 3.70, *p* < 0.01, d = 0.45). Moreover, the expected total control effect was also observed with participants recalling significantly more respond than suppress words (*M* = 91.76, *SD* = 13.05 vs. *M* = 74.90, *SD* = 19.33; *t* (86) = 6.11, *p* < 0.01, d = 1.02). These findings are entirely consistent with previous studies using the TNT task and show a robust forgetting effect of ~8% which is comparable to the negative control effects obtained in other TNT studies (typically between 6–9%; [1, 3, 37]).

**Fig 1:**
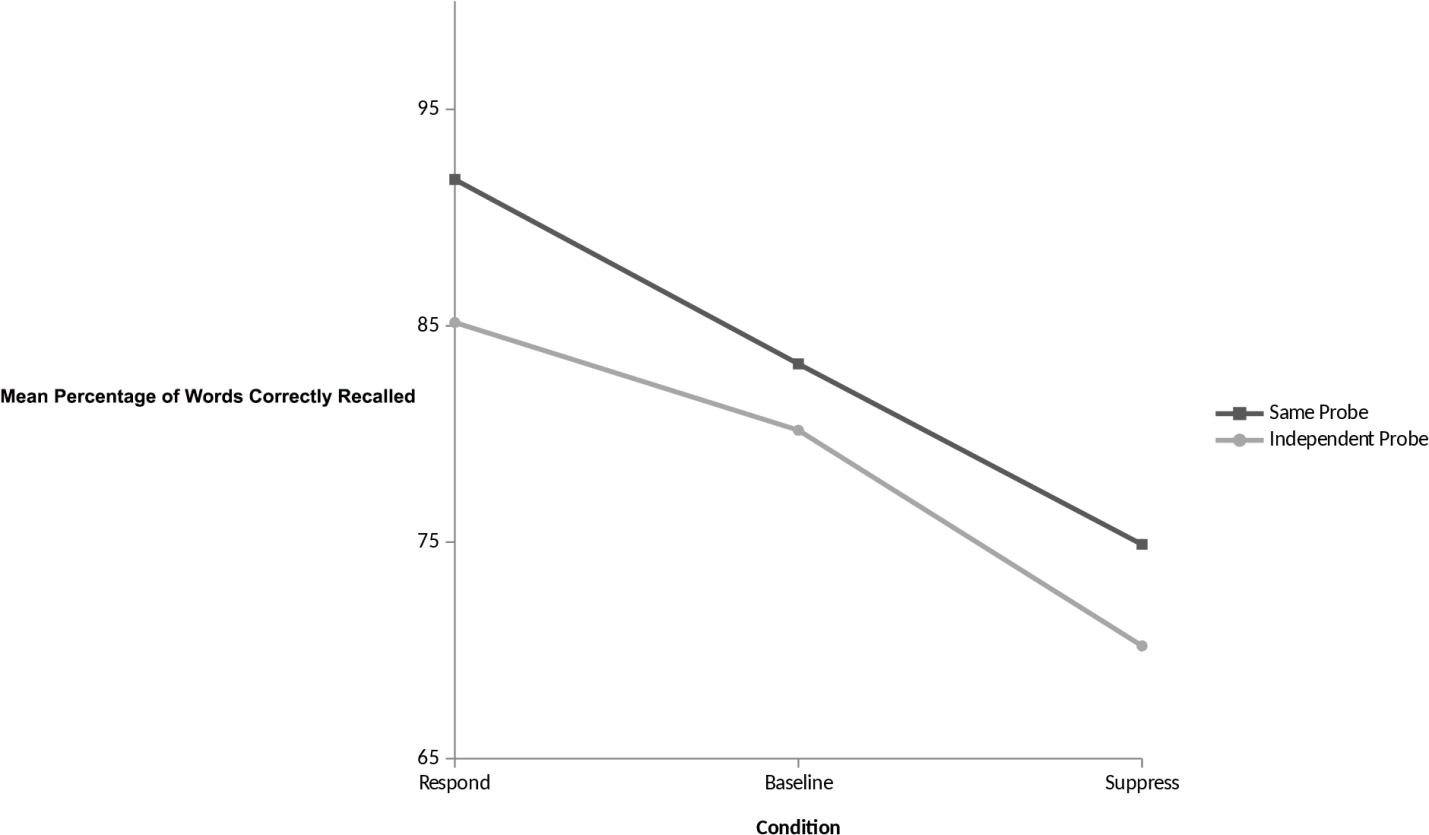
Mean percentage of words recalled as a function of instruction type on cued recall and independent probe tests of the think/no-think task.

A similar pattern emerged for the retrieval of items using independent probes at final test. A one-factor ANOVA showed a main effect of instruction, *F* (2, 84) = 17.88, *p* < 0.01 with subsequent pairwise analyses revealing that participants recalled significantly more respond than baseline words (*M* = 85.15, *SD* = 13.65 vs. *M* = 80.17, *SD* = 14.78; *t* (86) = 2.27, *p* < 0.03, d = 0.35), thereby indicating a positive control effect. Participants also showed a negative control effect whereby they recalled significantly fewer suppress words than baseline (*M* = 70.21, *SD* = 20.98 vs. *M* = 80.17, *SD* = 14.78; *t* (86) = 3.60, *p* < 0.01, d = 0.55). Moreover, the expected total control effect was also observed with participants recalling significantly more respond than suppress words (*M* = 85.15, *SD* = 13.65 vs. *M* = 70.21, *SD* = 20.98; *t* (86) = 5.36, *p* < 0.01, d = 0.84). See Fig 1. Again, these findings are consistent with studies which have reported cue-independent forgetting effects in the TNT task [1, 35, 36], and thereby contribute to the body of evidence which supports an inhibitory control framework of memory retrieval.

#### ‘Impossible’ retrieval practice task

**Fig 2:**
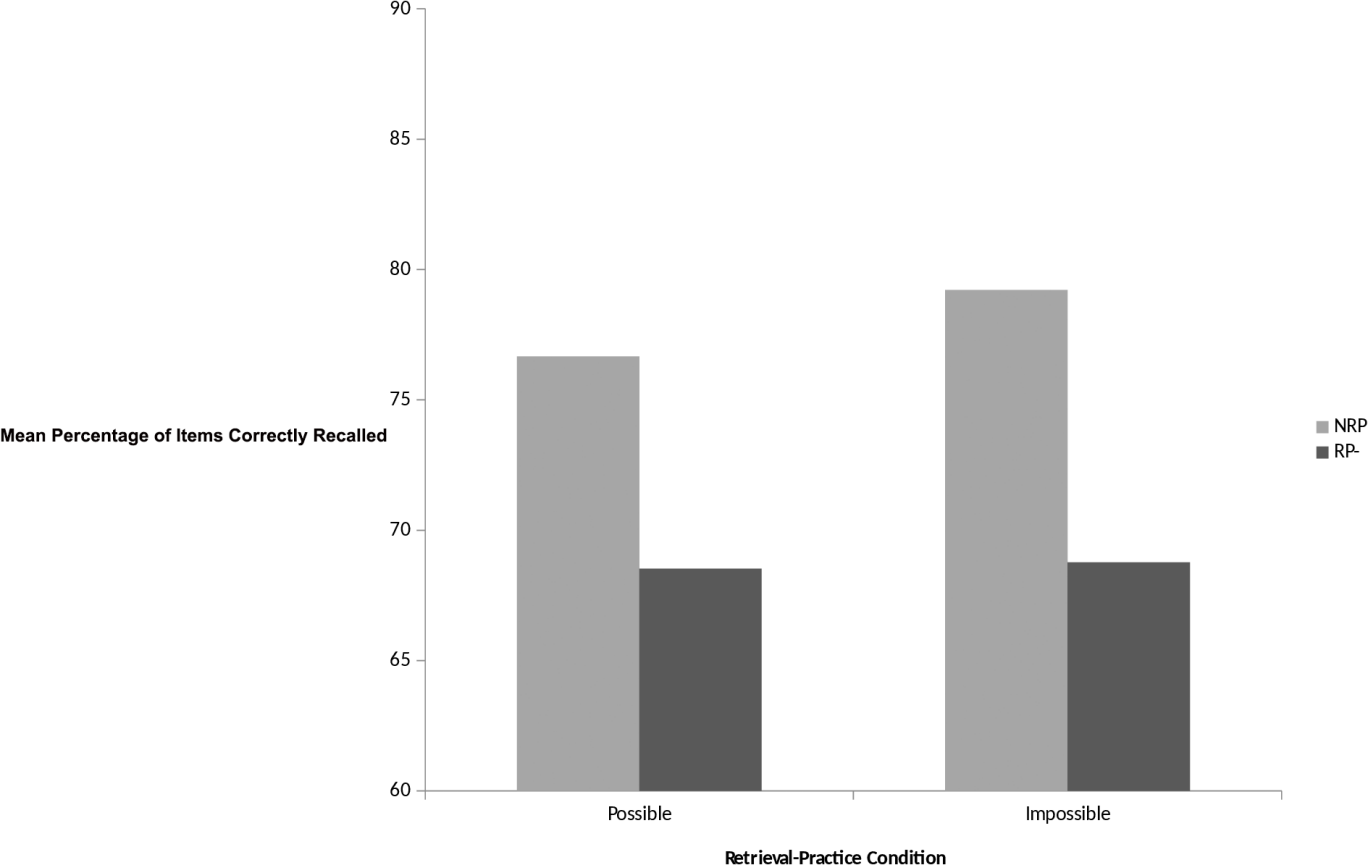
Mean percentage of words recalled as a function of retrieval-practice condition on the retrieval practice task.

The principal dependent measure of interest here is the proportion of Rp- and Nrp items correctly recalled from categories that received possible or impossible retrieval practice. This was analysed using a 2 (condition: Rp- vs. Nrp) x 2 (retrieval practice: possible vs. impossible) repeated measures ANOVA. This analysis revealed a significant retrieval induced forgetting effect whereby participants recalled significantly fewer Rp- items than Nrp items, *F* (1, 85) = 21.90, *p* < 0.01, d = 0.66; (*M* = 68.65, *SD* = 13.53 vs. *M* = 77.94, *SD* = 14.73). There was no effect of retrieval practice, or a condition by retrieval practice interaction; *F* (1, 85) = 0.61, *p* > 0.05, *F* (1, 85) = 0.41, *p* > 0.05, respectively. Furthermore, the size of the retrieval-induced forgetting effect in the possible retrieval practice condition was in the region of ~8% (Nrp *M* = 76.67, *SD* = 16.87 vs. Rp- *M* = 68.52, *SD* = 17.61), and in the impossible retrieval practice condition was in the region of ~10% (Nrp *M* = 79.21, *SD* = 15.08 vs. Rp- *M* = 68.77, *SD* = 17.20). See Fig 2.

The pattern of results obtained here is consistent with previous studies that have used this modified retrieval practice paradigm [34, 82]. Furthermore, the fact that our study failed to find a significant difference between possible and impossible retrieval practice is also consistent with previous research by Storm and colleagues. It is worth noting here, however, that our participants demonstrated elevated levels of recall for both baseline and Rp- items in comparison to previous work, which is likely to reflect differences in the materials employed. Nonetheless, the present findings show RIF effects that are independent of whether retrieval practice is possible or not and thus provide additional support for an inhibitory account of retrieval-induced forgetting [9, 49].

#### Recall during retrieval practice

During the possible retrieval practice phase we found that participants recalled 73% of the exemplars correctly. As none of the exemplars corresponded to a given category in the impossible retrieval practice phase, participants did not generate any of the exemplars correctly. Participants did, however, generate an incorrect exemplar 27% of the time in this condition. These incorrect responses either began with the first letter of the cue stem but not the second letter, or did not qualify as a member of the category. Taken together, our participants demonstrated robust forgetting effects on both retrieval tasks that purport to reflect inhibition. As such, these findings are consistent with a body of work that suggests that competition resolution and suppression efforts can result in diminished recall for to-be-forgotten material [15, 34–36, 40]. Furthermore, recall accuracy observed on both forgetting measures (i.e., TNT and retrieval practice) would suggest that there is nothing unusual about the group of participants used in the present study. Indeed, the level of retrieval performance is broadly comparable with other studies in the field which have explored inhibitory control using these paradigms. Moreover, the fact that the forgetting effects reported here have been found on measures that are commonly purported to distinguish between inhibitory and interference-based accounts (e.g., independent-probe test and the ‘impossible’ retrieval-practice task) would lend additional support to the idea that these measures provide a reliable index of memory inhibition.

### Comparison across memory inhibition tasks

In order to determine whether there were any significant relationships between the forgetting effects observed across both measures of memory inhibition, the size of the forgetting effect was calculated for each task. In line with Anderson and colleagues [3, 35], the size of the forgetting effect on the TNT task was calculated by subtracting the suppression score from the baseline score for both the same-probe and independent-probe tests, with higher scores reflecting a larger forgetting effect. Consistent with this, the size of the forgetting effect on the retrieval-practice task was calculated by subtracting the Rp- scores from baseline Nrp performance for both possible and impossible retrieval conditions, respectively.

Pearson correlational analyses revealed that there was a significant correlation between the extent of forgetting observed on the cued-recall and independent-probe test of the TNT task, *r* (87) = 0.40, *p*< 0.01. Our analyses also revealed that there was a significant correlation between the size of the forgetting effect observed on the possible retrieval practice task and the impossible retrieval practice task, *r* (87) = 0.36, *p* < 0.01.

Pearson correlations were also carried out *between* the forgetting effects observed on the TNT task and the retrieval-practice task. These revealed no significant correlations between the two tasks (possible Rp- and same-probe test, *r* (87) = 0.17, *p* > 0.05; possible Rp- and independent recall, *r* (87) = 0.12, *p* > 0.05; impossible Rp- and same-probe test, *r* (87) = 0.06, *p* > 0.05; impossible Rp- and independent test, *r* (87) = -0.14, *p* > 0.05). Thus, we can be reasonably confident that, on the basis of the measures used here, there is no evidence to support the notion that inhibitory performance on TNT and RIF is correlated.

Additionally, in order to establish reliability for the TNT task, we conducted a split-half analysis by separating the target words into two lists. Pearson correlations indicated that performance on the same-probe test for list 1 was correlated to recall on the independent-test for list 2, r (87) = 3.75, p < 0.01. Moreover, we also found that performance on the same-probe test for list 2 was correlated with recall on the independent-probe test for list 1, r (87) = 44, p < 0.01. These findings are consistent with our overall findings from the same- and independent-probe tests and would indicate that the reliability of the tests employed here is robust and therefore unlikely to have contributed to the absence of a correlation between the two measures of inhibitory forgetting.

### Behavioural inhibition measures

Pearson correlations between performance on behavioural response measures on the Stroop and the Go/No-Go tasks revealed that some measures of the Stroop and the GNGT tasks were correlated. For example, Stroop facilitation was correlated with the RT for go trials, and the number of Stroop errors made was also correlated with the number of no-go errors made. The fact that the level of errors made on both tasks was correlated provides support that the errors made on both tasks reflect an inability to inhibit prepotent responses. See Table 1 for correlations.

**Table 1:** Pearson correlation coefficients between memory inhibition measures of think/no-think and retrieval practice and behavioural inhibition measures of Stroop and Go/No-Go.

	Same Probe Test	Independent Probe Test	Possible Rp	Impossible Rp	Stroop Interference	Stroop Facilitation	Stroop Errors	No-go Errors	Go Errors	RT for go trials
**Same Probe Test**		.396**	.174	- .063	.029	-.036	.006	.032	-.057	-.155
**Independent Probe Test**			.120	-.135	.019	-.041	.074	.002	.033	.084
**Possible Rp**				.363**	.063	.054	-.010	-.037	.162	-.065
**Impossible Rp**					.030	.030	-.141	-.068	.055	-.048
**Stroop Interference**						-.061	.159	.021	.060	-.110
**Stroop Facilitation**							-.069	.049	.043	-.304**
**Stroop Errors**								.244*	-.100	-.161
**No-Go Errors**									-.033	-.037
**Go Errors**										.062
**RT for go trials**										

* = p < 0.05

** = p < 0.01

It is important to mention here, however, that a number of measures were not found to be correlated between these two tasks, including Stroop interference and no-go errors. The relationship between the GNGT and the Stroop task has been studied by several researchers and only weak correlations have emerged between interference on the Stroop task and performance on the GNGT [85, 86]. This suggests that Stroop and GNGT tasks have relatively few commonalities and may primarily be assessing different psychological processes.

Our findings also lend support to a growing body of research which suggests that complex executive tasks, including those tasks that are frequently used to tap into inhibition-related functions tend to show poor reliability [87, 88]. Although the origin of these low reliabilities remains unclear, they are likely to be multi-faceted. One possibility is that measures of executive functions are only valid when they pose significant attentional control demands [88]. It is possible that, as individuals gain more experience on a task and develop strategies to cope with task demands, less attentional control is required leading to low reliability between measures.

Another possibility is that both the Stroop and Go/No-Go tasks rely on different methods for inducing cognitive conflict. It is possible that these different forms of conflict may lead to different neural mechanisms being activated. For example, in the Stroop task, conflict is produced by competing stimuli and therefore a mechanism that filters out distracting visual information may be useful. However, this same mechanism may not be relevant for the Go/No-Go task in which there are no distractors. Furthermore, whilst the GNGT requires participants to hold only one target stimulus in mind and relies on short-term memory to remember the stimulus for a fixed period of time without actively manipulating the information, the Stroop task requires participants to focus on novel information in each trial and arguably involves greater executive control resources. Thus, it is possible that whilst the GNGT assesses the ability to inhibit the execution of a behavioural response, the Stroop assesses the ability to exert inhibition or interference control in higher cognitive tasks that involve working memory and flexible set-shifting. Our results suggest that while there may be some commonalities, the Stroop and GNGT tasks may tap into different aspects of selective attention and response inhibition. Both tests are therefore important and useful in the multifaceted assessment of behavioural inhibition. See S1 Table for a summary of participant’s performance across all tasks.

### Relationship between memory inhibition and behavioural inhibition

Pearson correlations revealed that the forgetting effects on the TNT task were not significantly correlated with performance on either the Stroop or the Go/No-Go task (see Table 1 for correlations). Similarly, Pearson correlations also revealed that the forgetting effects on the retrieval practice task were not significantly correlated with performance on either the Stroop task or the Go/No-Go task (see Table 1 for correlations) (Participants raw data across memory and behavioural tasks in supplementary information section).

## Discussion

The main finding to emerge from the present study is that, while our participants produced reliable forgetting effects that were consistent with an inhibitory account in both TNT and ‘impossible’ retrieval-practice tasks, participants’ performance across memory tasks was not correlated; that is, the extent of forgetting on one inhibitory task was not reflected in the level of forgetting observed on another. Notwithstanding the fact that there are marked differences in inhibitory control between individuals, one could still have expected performance to have been correlated. Moreover, our measures of memory inhibition were uncorrelated with recognised measures of behavioural inhibition. Assuming that each of these tasks do indeed reflect inhibitory control, our findings could be taken as a challenge to the notion of a single inhibitory mechanism. Indeed, the assumed relationship regarding inhibitory control across various inhibitory tasks may represent an overly simplistic view of what we mean by cognitive inhibition and, if we are to progress our understanding of inhibition and how it works, we may need to revisit some of these basic underlying assumptions.

In saying this, however, a null finding is not necessarily evidence for a lack of a relationship. Thus, we need to consider whether our study represents a good test of the relationship between the various measures of inhibition employed [89]. In this regard, it is important to mention here that each test provided in its own right a recognised measure of inhibition. Indeed, if we were to take each test in isolation, our findings provide support for an inhibitory account of forgetting and are consistent with a large body of research [1, 3, 4]. It is only when we explore the relationship between performance on the memory measures, or with measures of behavioural inhibition that our findings deviate from what could be expected. As this is the first study to explore the relationship between memory and behavioural inhibition across a variety of tasks, it may be tempting to dismiss these findings as spurious. While we advise caution as to how our results are ultimately interpreted, we believe that the absence of any predicted relationships between tests–following very careful individual test methodology–poses a challenge to classical views surrounding inhibition. In particular, we believe that our study serves to demonstrate the need for a much more sophisticated understanding of the relationship between inhibition and the demands posed by particular tasks.

Our study is notable not only because it failed to elicit significant correlations between recognised measures of memory inhibition, but also with recognised measures of behavioural inhibition. This is in contrast to other published studies which have reported a relationship between behavioural inhibition (as indexed by the stop signal task) and memory inhibitory forgetting on both the retrieval-practice task [52] and the TNT task [50, 51, 53]. It is worth noting, however, that these particular studies employed only the stop signal task as an index of behavioural inhibition. Logically, therefore, there is the possibility that the apparent disparity between these studies and the current study may be due to some other component and not inhibition per se. Additionally, the fact that we did not use the stop signal task and instead used different behavioural inhibition tasks as a means of building upon existing research and providing a multifaceted approach to studying the relationship between memory and behavioural inhibition, it is possible that the type of inhibition evoked across tasks may have differed and may therefore be unrelated to the inhibition indexed by the memory tasks. This possibility highlights the need for much more specificity about what kind of inhibition is invoked under particular task conditions. At the present, this field of research does not permit this level of precision.

Also, the fact that we failed to find a significant correlation between performance on our memory inhibition tasks and the GNGT may seem problematic given that both GNGT and stop signal tasks tend to be used interchangeably. Indeed, it is widely assumed that the same inhibitory processes are responsible for cancelling a response [90–92]. More recent research, however, has indicated that this may not necessarily be the case [93]. As the GNGT involves the instruction ‘not to go’ being given before any action is undertaken and the stop signal task involves the ‘go response’ being issued prior to the ‘no go’ instruction, both tasks differ in how behavioural inhibition is achieved with the GNGT more likely to involve automatic inhibition and the stop signal task more likely to involve controlled inhibition [93]. Thus, individuals with deficits in controlled inhibition are more likely to demonstrate impairments in the stop signal task rather than the GNGT task. This is consistent with research that has found group differences in the stop signal task but not in the GNGT task [94, 95]. Taken together, these findings would indicate that memory inhibition may not be related to automatic behavioural inhibition but rather controlled behavioural inhibition.

We also need to consider the possibility that the failure to find a significant relationship between performance on these particular tasks may have been due to the possibility that some of these tasks could have tapped into inhibitory processes in only a limited way. In other words, it would be simple to dismiss the absence of such relationships if one could demonstrate that we had not obtained as ‘pure’ a measure of inhibition as possible. This is an issue for any researcher interested in inhibitory control as it is widely acknowledged that non-inhibitory mechanisms such as associative interference and blocking have the potential to contribute to the forgetting effects typically observed [4, 37]. For example, competition from strong items and the resultant blocking of weaker items on the retrieval-practice task [96], and thought substitution on the TNT task can be potent non-inhibitory contributors to observed forgetting effects [97–100].

We do not believe that this is an issue in the current study, however. The battery of forgetting tasks used in the present study employed widely recognised means of ensuring ‘pure’ measures of inhibition. More specifically, for the TNT task, we incorporated the use of independent probes at final test [9, 11, 35, 49] (although see [31, 32] for an alternative theoretical perspective). The fact that forgetting effects persisted despite the use of independent probes is consistent with an inhibitory account. Similarly, our choice of the adapted version of the retrieval-practice task [34] allowed us to infer that, as far as possible, any observed forgetting effect could be attributed to inhibition. According to non-inhibitory accounts of forgetting, forgetting reflects changes in the relative strength of practised and non-practised items [55, 96]. Given that the version of the retrieval-practice task adopted here did not actually involve the practice of any target items (although we accept the possibility that some participants may have covertly retrieved items which they did not report), there was little means by which items could be strengthened and, by the same token, other memories weakened. Thus, if retrieval-induced forgetting was simply a function of non-inhibitory mechanisms, no forgetting should have emerged in our retrieval practice task. The fact that forgetting effects emerged even when retrieval-practice was deemed impossible indicates that the observed forgetting effects were consistent with an inhibitory account. Taken together, the paradigms adopted in this study would seem to have produced as ‘pure’ a measure of memory inhibition as is currently possible. In other words, we can be reasonably confident that the memory tasks used in the present study were tapping into inhibition.

We also need to consider the possibility that the absence of significant correlations between memory inhibition tasks may have had something to do with their test-retest reliability. If the tests employed were inherently unreliable then it would be unsurprising that no relationships emerged between tests. We are confident, however, that this is unlikely to have been the case here. In our impossible retrieval task procedure, we found that both possible and impossible retrieval practice performance was correlated. This finding is consistent with previous work [34, 82] suggesting that the paradigm has good test-retest reliability. In order to establish reliability for the TNT task, we also conducted a split-half analysis by separating target words into two lists. Pearson correlations indicated that performance on the same-probe test for list 1 was correlated with recall on the independent-test for list 2. Moreover, we also found that performance on the same-probe test for list 2 was correlated with recall on the independent-probe test for list 1. These findings are consistent with our overall findings from the same- and independent-probe tests. They are also consistent with a recent study which found that initial suppression performance predicts long-term suppression effects [7]. Taken together, these findings would indicate that the lack of a correlation between retrieval-induced forgetting and suppression-induced forgetting on the retrieval-practice and TNT tasks respectively, is unlikely to have been due to poor test-retest reliability.

Lastly, we need to acknowledge that our study failed to find a significant correlation between participants’ performance on measures of Stroop interference or the Go/No-Go task with the magnitude of forgetting observed on either the TNT or retrieval-practice task. Again, it is important to consider the task demands associated with particular paradigms and the extent to which these may mask common features of inhibition [101, 102]. As mentioned earlier, measures of executive functioning are only reliable when they pose significant attentional demands [88]. Therefore, as more experience is gained on a task, individuals may develop new strategies to cope with the particular demands of the task, thereby resulting in low reliability between measures.

In order to address this task specific variance issue, future research needs to develop more sophisticated approaches in order to add further confidence to inhibitory interpretations. For example, using multiple variants of the same task [102, 103] may prove to be one means of dealing with this impurity problem. The strategy of keeping the tasks as similar as possible, however, is likely to prove problematic because the resulting common variance comes from both inhibitory demands and other demands related to the methodology. Furthermore, this strategy does not alleviate reliability and construct validity problems. Setting aside these potential contributors to the absence of a clear relationship between memory inhibition and behavioural inhibition, our findings can be interpreted as being consistent with the notion of a flexible control framework [49]. This theoretical perspective proposes that the failure to find a significant relationship between an individual’s ability to inhibit across tasks may reflect the unique task properties of both paradigms and the diverse range of contexts which give rise to inhibitory effects. For example, forgetting effects in the TNT task arise as a consequence of suppressing a highly trained memory when repeatedly confronted with a reminder whilst, in the retrieval-practice paradigm, forgetting is essentially a by-product of attempting to resolve competition.

Furthermore, the TNT task involves an explicit demand to ‘not think’ about particular information that may interfere with the recall of other learned information whereas the retrieval-practice task involves inhibitory effects being induced unintentionally. Thus, given that there are fundamental differences in the way in which inhibition is evoked, it is possible that both tasks place different demands on inhibitory control and thus reflect different forms of inhibition. The fact that our findings failed to show any significant relationship between these various inhibitory may therefore suggest that the inhibition involved in each task may not be the same.

These findings challenge much of the current thinking about inhibitory control as a common inhibitory process [12, 21, 38, 63]. Rather, our findings suggest that the recruitment of inhibitory control on these various tasks may depend upon the differing properties of each task and differing task demands. Future research in this field needs to establish if this is the case. This will only be possible by generating specific predictions based on the demands of particular inhibitory tasks.

In conclusion, using a within-subjects design, our study attempted to consolidate inhibition findings across a range of tasks which are assumed to access inhibitory control. While we found evidence of successful forgetting on both forgetting tasks consistent with an inhibitory account, no significant correlations emerged between the level of forgetting on one task with forgetting on another. We also found no evidence of a relationship between behavioural response measures and forgetting tasks. We believe that the failure to find any such relationship poses a challenge to some of the current assumptions about cognitive inhibition and should prompt us to consider what we actually mean by inhibition. What is clear is that inhibition for goal-driven control is most likely a latent variable that has varying constructs and semantic definitions. Thus, the challenge for future researchers in this field is to address the possibility that inhibitory control may not be a unitary process and that, even if it is, we need to consider more closely the cognitive demands of particular tasks and how these may affect our interpretation of inhibitory forgetting effects.

One of the dangers in the cognitive literature at the moment is that when relationships are found between various tests of inhibition, this is taken as support for the existence of a single inhibitory mechanism. When no such relationships are found, however, the assumption is made that inhibition is there but, because of task demands, the relationship could not been demonstrated. While we acknowledge that inhibitory demands are likely to vary between tasks, we have yet to establish empirically that such task demands themselves are responsible for the poor relationships across inhibitory tasks. Until we do so, it may be fair for detractors to say that such a theoretical position is an instance of ‘having one’s cake and eating it’. Thus, if we are to progress our understanding of cognitive inhibition, we need to adopt a much more nuanced understanding of what these various tasks demands might be in order to generate specific predictions that are testable. Without such a level of specificity, the arguments between inhibitory theorists and their detractors will continue.

## Supporting Information

S1 Table Participants raw data across memory and behavioural tasks. (DOCX)
