# Supplementary material for: What Do We Really Know about Cognitive Inhibition? Task Demands and Inhibitory Effects across a Range of Memory and Behavioural Tasks
Source: PLoS One. 2015 Aug 13;10(8):e0134951. doi: 10.1371/journal.pone.0134951 (PMC4536050; doi:10.1371/journal.pone.0134951)
Supplement: S1 Table — (DOCX) [file pone.0134951.s001.docx]

**S1 Table. Raw Values for Behavioural and Memory Inhibition Measures.**

| **No.** | **Stroop Interference** | **Stroop facilitation** | **Stroop Incongruent Eroors** | **GNGT- No-go Errors** | **GNGT- Go Errors** | **GNGT- Go Reaction Time** | **Same Probe Forgetting Effect** | **Independent Test Forgetting Effect** | **Possible Forgetting Effect** | **Impossible Forgetting Effect** |
| --- | --- | --- | --- | --- | --- | --- | --- | --- | --- | --- |
| **1** | .7078 | .1095 | 6.00 | 10.00 | 2.00 | 295.56 | 25.00 | 16.66 | 25.00 | 8.33 |
| **2** | .2248 | .0913 | 4.00 | 6.00 | 2.00 | 235.12 | 41.66 | 8.33 | 33.33 | 8.34 |
| **3** | .3525 | .0109 | 3.00 | .00 | 8.00 | 254.09 | -16.66 | .00 | 41.67 | 16.66 |
| **4** | .1624 | -.0431 | .00 | 9.00 | .00 | 280.29 | 8.33 | -33.33 | -16.66 | 16.66 |
| **5** | .2757 | -.0384 | 3.00 | 11.00 | 5.00 | 319.85 | 25.00 | 50.00 | -25.00 | -8.33 |
| **6** | .1972 | .0201 | 1.00 | 5.00 | .00 | 235.72 | 8.34 | -8.33 | 33.33 | 33.33 |
| **7** | .2372 | -.0343 | 4.00 | 6.00 | 9.00 | 311.29 | 8.33 | 16.67 | -8.33 | 16.67 |
| **8** | .1037 | .0245 | .00 | 5.00 | .00 | 323.33 | .00 | .00 | -33.33 | -33.33 |
| **9** | .2776 | -.0125 | 2.00 | 4.00 | 5.00 | 304.08 | .00 | -41.67 | 8.34 | 8.34 |
| **10** | .7078 | .1095 | 3.00 | 8.00 | 3.00 | 327.05 | 25.00 | 16.66 | 8.33 | 8.33 |
| **11** | .2248 | .0913 | 5.00 | 9.00 | 4.00 | 263.41 | 41.66 | 8.33 | 16.67 | 16.67 |
| **12** | .2425 | -.0675 | 3.00 | .00 | 2.00 | 317.31 | -41.67 | -33.33 | .00 | 16.66 |
| **13** | .4126 | -.1227 | 5.00 | .00 | 2.00 | 335.45 | -16.66 | .00 | 41.67 | 16.66 |
| **14** | .6863 | .1095 | 6.00 | .00 | 2.00 | 263.41 | 33.34 | 41.66 | .00 | -33.33 |
| **15** | .2372 | -.0050 | 5.00 | 2.00 | 8.00 | 298.07 | -16.66 | .00 | 41.67 | 33.33 |
| **16** | .6863 | -.1658 | 2.00 | 5.00 | 5.00 | 270.93 | -16.66 | 41.67 | .00 | .00 |
| **17** | .2776 | .0012 | .00 | .00 | .00 | 304.08 | 8.34 | -8.33 | .00 | .00 |
| **18** | .7078 | .1140 | .00 | 3.00 | .00 | 327.05 | 8.33 | 16.67 | .00 | .00 |
| **19** | .1402 | -.0231 | 2.00 | 4.00 | .00 | 273.94 | 25.00 | 16.66 | 25.00 | 25.00 |
| **20** | .1037 | .0948 | 8.00 | 7.00 | .00 | 295.56 | 41.66 | 8.33 | 8.34 | 8.34 |
| **21** | .6863 | .2055 | .00 | 3.00 | 5.00 | 235.12 | 16.67 | 8.33 | 33.33 | 33.33 |
| **22** | .2600 | .0848 | 5.00 | 8.00 | 4.00 | 277.68 | 25.00 | 50.00 | -33.33 | .00 |
| **23** | .4396 | .0245 | .00 | .00 | 8.00 | 304.08 | .00 | 8.33 | 41.67 | 8.34 |
| **24** | .1402 | .0880 | .00 | 3.00 | 9.00 | 327.05 | 16.66 | 8.33 | 25.00 | 33.33 |
| **25** | .1782 | .0626 | .00 | 3.00 | .00 | 392.16 | -16.66 | 41.67 | .00 | .00 |
| **26** | .0941 | .0234 | .00 | .00 | 5.00 | 319.25 | .00 | .00 | 41.67 | 41.67 |
| **27** | .6863 | .0880 | .00 | 3.00 | .00 | 277.68 | .00 | 8.33 | .00 | .00 |
| **28** | .5076 | -.0393 | 4.00 | 15.00 | 8.00 | 283.38 | 16.67 | 8.33 | 33.33 | 33.33 |
| **29** | .7078 | -.0393 | 2.00 | .00 | 3.00 | 229.90 | .00 | .00 | -50.00 | 25.00 |
| **30** | .4313 | .0848 | 2.00 | 12.00 | 10.00 | 270.93 | -16.67 | -25.00 | .00 | .00 |
| **31** | .1037 | .0245 | 6.00 | 9.00 | .00 | 263.41 | -33.33 | -8.33 | -41.67 | -33.33 |
| **32** | .2776 | -.0125 | .00 | .00 | 1.00 | 273.94 | .00 | -41.67 | .00 | -16.66 |
| **33** | .1349 | .0199 | 2.00 | 9.00 | 1.00 | 298.49 | .00 | .00 | .00 | -8.33 |
| **34** | .2425 | -.0675 | 4.00 | 6.00 | 5.00 | 277.68 | 33.34 | 16.66 | -7.67 | 50.00 |
| **35** | .6863 | .0880 | 6.00 | 18.00 | 3.00 | 283.38 | .00 | .00 | 16.67 | .00 |
| **36** | .5076 | -.0393 | 2.00 | 1.00 | 2.00 | 229.90 | 16.67 | 8.33 | .00 | 33.33 |
| **37** | .2600 | -.0231 | 1.00 | 7.00 | 3.00 | 287.36 | 33.33 | 8.34 | 41.67 | 16.67 |
| **38** | .4396 | .0948 | 3.00 | 5.00 | 7.00 | 298.07 | 25.00 | 50.00 | 8.34 | -16.66 |
| **39** | .1402 | .2055 | 2.00 | 7.00 | 3.00 | 270.93 | .00 | 8.33 | 16.66 | 41.67 |
| **40** | .1343 | .0316 | 1.00 | 13.00 | 16.00 | 275.78 | 16.66 | 8.33 | 25.00 | .00 |
| **41** | .0403 | .0378 | .00 | 5.00 | .00 | 270.18 | 25.00 | 33.33 | .00 | 8.33 |
| **42** | .2600 | -.0231 | 2.00 | .00 | 9.00 | 273.94 | 33.33 | 8.34 | .00 | 8.34 |
| **43** | .4396 | .0948 | .00 | .00 | 15.00 | 287.52 | 25.00 | 50.00 | -33.33 | -16.66 |
| **44** | .1402 | .2055 | .00 | 8.00 | .00 | 255.55 | .00 | 8.33 | .00 | 25.00 |
| **45** | .1681 | .1140 | 8.00 | .00 | 1.00 | 198.65 | 8.33 | 33.34 | 16.67 | -8.33 |
| **46** | .5504 | -.0050 | 2.00 | .00 | 2.00 | 340.81 | .00 | .00 | -50.00 | 8.33 |
| **47** | .4313 | .0848 | .00 | .00 | 3.00 | 323.33 | 25.00 | 33.33 | .00 | 8.33 |
| **48** | .1037 | -.0125 | .00 | .00 | 16.00 | 327.05 | 8.33 | 50.00 | 33.33 | 16.67 |
| **49** | .2776 | .0913 | .00 | 5.00 | 1.00 | 235.12 | 8.33 | 33.34 | 16.67 | 41.67 |
| **50** | .2600 | .0199 | .00 | .00 | 3.00 | 287.36 | -41.67 | -33.33 | .00 | 33.33 |
| **51** | .1260 | -.0675 | .00 | .00 | 8.00 | 304.08 | 25.00 | 33.33 | .00 | -8.33 |
| **52** | .1597 | -.1227 | 1.00 | 4.00 | .00 | 327.05 | 8.33 | -33.33 | 16.67 | 33.33 |
| **53** | .1037 | .0848 | .00 | .00 | .00 | 263.41 | 25.00 | 50.00 | 33.33 | -8.33 |
| **54** | .5076 | -.1227 | 3.00 | .00 | 3.00 | 233.59 | 33.33 | 8.34 | 16.67 | 16.67 |
| **55** | .6863 | .4126 | .00 | .00 | 5.00 | 247.66 | 8.33 | -33.33 | 33.33 | 33.33 |
| **56** | .0848 | .4313 | .00 | 3.00 | 4.00 | 278.45 | 25.00 | 50.00 | 25.00 | 25.00 |
| **57** | .2776 | .1402 | .00 | .00 | 7.00 | 266.54 | 66.66 | -41.67 | .00 | 16.67 |
| **58** | .5504 | -.0050 | 5.00 | 9.00 | 4.00 | 323.33 | -25.00 | -16.66 | -8.33 | 16.66 |
| **59** | .4126 | -.1227 | 1.00 | .00 | .00 | 298.07 | 25.00 | 16.66 | 16.67 | 58.34 |
| **60** | .0350 | -.0030 | .00 | 3.00 | 2.00 | 272.28 | 8.33 | 50.00 | 33.33 | .00 |
| **61** | .4389 | -.1658 | 4.00 | 2.00 | 1.00 | 327.63 | 33.34 | 41.66 | .00 | .00 |
| **62** | .2399 | .0012 | .00 | 13.00 | 3.00 | 340.81 | 8.33 | 33.34 | 16.67 | -8.33 |
| **63** | .1681 | .1140 | .00 | 2.00 | 3.00 | 317.31 | .00 | .00 | -50.00 | 8.33 |
| **64** | .0689 | .0223 | .00 | .00 | 8.00 | 335.45 | -41.67 | -33.33 | .00 | 16.66 |
| **65** | .1260 | .0932 | 1.00 | 5.00 | 6.00 | 309.03 | -8.33 | 8.33 | 16.66 | 16.66 |
| **66** | .1597 | -.0212 | .00 | .00 | 6.00 | 319.98 | .00 | -16.67 | -33.33 | -33.33 |
| **67** | .0350 | -.0030 | 2.00 | .00 | 10.00 | 309.03 | 16.66 | 8.33 | -16.66 | 8.34 |
| **68** | .4389 | -.1658 | 4.00 | .00 | .00 | 319.98 | 8.33 | 50.00 | 33.33 | .00 |
| **69** | .2399 | .0012 | .00 | .00 | 3.00 | 323.56 | 33.34 | 41.66 | .00 | .00 |
| **70** | .4126 | .0245 | .00 | .00 | 7.00 | 277.68 | 8.33 | -33.33 | 16.67 | 16.66 |
| **71** | .4313 | .0880 | .00 | 2.00 | 3.00 | 304.08 | 16.66 | 8.33 | 25.00 | 33.33 |
| **72** | .2248 | .0223 | 3.00 | .00 | 9.00 | 298.07 | .00 | 25.00 | 41.67 | 41.67 |
| **73** | .5076 | .0109 | 5.00 | .00 | 6.00 | 327.05 | 16.67 | .00 | 41.67 | 41.67 |
| **74** | .2600 | .0378 | 6.00 | 3.00 | 6.00 | 263.41 | 33.33 | 8.33 | 16.67 | 16.67 |
| **75** | .4396 | -.0393 | 7.00 | 4.00 | 5.00 | 283.38 | -33.33 | -8.33 | -33.33 | -33.33 |
| **76** | .6863 | -.0125 | .00 | 3.00 | 15.00 | 283.38 | 8.33 | 50.00 | 33.33 | 16.67 |
| **77** | .2776 | .1095 | 5.00 | 3.00 | 3.00 | 273.94 | 33.34 | 41.66 | 33.33 | -33.33 |
| **78** | .7078 | .0913 | 4.00 | 2.00 | 4.00 | 272.52 | 8.33 | 33.34 | -8.33 | 41.67 |
| **79** | .2248 | -.0393 | 2.00 | .00 | 8.00 | 323.33 | .00 | .00 | 33.33 | -8.33 |
| **80** | .4126 | -.0231 | 4.00 | 4.00 | 9.00 | 256.66 | -41.67 | -33.33 | -8.33 | -16.67 |
| **81** | .4313 | .5504 | 2.00 | 4.00 | 15.00 | 287.36 | -16.66 | .00 | 33.33 | 33.33 |
| **82** | .1037 | .2425 | 3.00 | 5.00 | 3.00 | 323.23 | 25.00 | 33.33 | 25.00 | 25.00 |
| **83** | .0245 | .1037 | 4.00 | .00 | 2.00 | 295.56 | -16.66 | 41.67 | .00 | .00 |
| **84** | .0880 | .6863 | 2.00 | 5.00 | 1.00 | 235.12 | .00 | -16.67 | .00 | .00 |
| **85** | -.0125 | .5076 | 1.00 | 3.00 | 4.00 | 244.58 | .00 | .00 | -33.33 | .00 |
| **86** | .1095 | .2600 | 5.00 | 2.00 | 2.00 | 298.25 | .00 | .00 | -16.66 | 33.33 |
| **87** | .6863 | .4396 | 3.00 | 1.00 | 5.00 | 250.23 | 16.67 | 8.33 | 41.67 | -16.66 |

**Raw Data for Memory Measures.**

| **No.** |  |  |  |  |  |  |
| --- | --- | --- | --- | --- | --- | --- |
| **1** |  |  |  |  |  |  |
| **2** |  |  |  |  |  |  |
| **3** |  |  |  |  |  |  |
| **4** |  |  |  |  |  |  |
| **5** |  |  |  |  |  |  |
| **6** |  |  |  |  |  |  |
| **7** |  |  |  |  |  |  |
| **8** |  |  |  |  |  |  |
| **9** |  |  |  |  |  |  |
| **10** |  |  |  |  |  |  |
| **11** |  |  |  |  |  |  |
| **12** |  |  |  |  |  |  |
| **13** |  |  |  |  |  |  |
| **14** |  |  |  |  |  |  |
| **15** |  |  |  |  |  |  |
| **16** |  |  |  |  |  |  |
| **17** |  |  |  |  |  |  |
| **18** |  |  |  |  |  |  |
| **19** |  |  |  |  |  |  |
| **20** |  |  |  |  |  |  |
| **21** |  |  |  |  |  |  |
| **22** |  |  |  |  |  |  |
| **23** |  |  |  |  |  |  |
| **24** |  |  |  |  |  |  |
| **25** |  |  |  |  |  |  |
| **26** |  |  |  |  |  |  |
| **27** |  |  |  |  |  |  |
| **28** |  |  |  |  |  |  |
| **29** |  |  |  |  |  |  |
| **30** |  |  |  |  |  |  |
| **31** |  |  |  |  |  |  |
| **32** |  |  |  |  |  |  |
| **33** |  |  |  |  |  |  |
| **34** |  |  |  |  |  |  |
| **35** |  |  |  |  |  |  |
| **36** |  |  |  |  |  |  |
| **37** |  |  |  |  |  |  |
| **38** |  |  |  |  |  |  |
| **39** |  |  |  |  |  |  |
| **40** |  |  |  |  |  |  |
| **41** |  |  |  |  |  |  |
| **42** |  |  |  |  |  |  |
| **43** |  |  |  |  |  |  |
| **44** |  |  |  |  |  |  |
| **45** |  |  |  |  |  |  |
| **46** |  |  |  |  |  |  |
| **47** |  |  |  |  |  |  |
| **48** |  |  |  |  |  |  |
| **49** |  |  |  |  |  |  |
| **50** |  |  |  |  |  |  |
| **51** |  |  |  |  |  |  |
| **52** |  |  |  |  |  |  |
| **53** |  |  |  |  |  |  |
| **54** |  |  |  |  |  |  |
| **55** |  |  |  |  |  |  |
| **56** |  |  |  |  |  |  |
| **57** |  |  |  |  |  |  |
| **58** |  |  |  |  |  |  |
| **59** |  |  |  |  |  |  |
| **60** |  |  |  |  |  |  |
| **61** |  |  |  |  |  |  |
| **62** |  |  |  |  |  |  |
| **63** |  |  |  |  |  |  |
| **64** |  |  |  |  |  |  |
| **65** |  |  |  |  |  |  |
| **66** |  |  |  |  |  |  |
| **67** |  |  |  |  |  |  |
| **68** |  |  |  |  |  |  |
| **69** |  |  |  |  |  |  |
| **70** |  |  |  |  |  |  |
| **71** |  |  |  |  |  |  |
| **72** |  |  |  |  |  |  |
| **73** |  |  |  |  |  |  |
| **74** |  |  |  |  |  |  |
| **75** |  |  |  |  |  |  |
| **76** |  |  |  |  |  |  |
| **77** |  |  |  |  |  |  |
| **78** |  |  |  |  |  |  |
| **79** |  |  |  |  |  |  |
| **80** |  |  |  |  |  |  |
| **81** |  |  |  |  |  |  |
| **82** |  |  |  |  |  |  |
| **83** |  |  |  |  |  |  |
| **84** |  |  |  |  |  |  |
| **85** |  |  |  |  |  |  |
| **86** |  |  |  |  |  |  |
| **87** |  |  |  |  |  |  |
